# Integrated meta-omics approaches reveal *Saccharopolyspora* as the core functional genus in *huangjiu* fermentations

**DOI:** 10.1038/s41522-023-00432-1

**Published:** 2023-09-19

**Authors:** Shuangping Liu, Zhi-Feng Zhang, Jieqi Mao, Zhilei Zhou, Jing Zhang, Caihong Shen, Songtao Wang, Maria L. Marco, Jian Mao

**Affiliations:** 1 National Engineering Laboratory for Cereal Fermentation and Food Biomanufacturing, School of Food Science and Technology, Jiangnan University, Wuxi, Jiangsu 214122 China; 2 Southern Marine Science and Engineering Guangdong Laboratory (Guangzhou), Guangzhou, 511458 China; 3 Shaoxing Key Laboratory of Traditional Fermentation Food and Human Health, Jiangnan University (Shaoxing) Industrial Technology Research Institute, Shaoxing, Zhejiang, 312000 China; 4 National Engineering Research Center of Huangjiu, Zhejiang Guyuelongshan Shaoxing Wine CO., LTD, Shaoxing, Zhejiang 312000 China; 5 Department of Food Science and Technology, National University of Singapore, Science Drive 2, 117542 Singapore, Singapore; 6 National Engineering Research Center of Solid-State Brewing, Luzhou, China; 7 Department of Food Science and Technology, University of California, Davis, CA USA

**Keywords:** Food microbiology, Applied microbiology

## Abstract

Identification of the core functional microorganisms in food fermentations is necessary to understand the ecological and functional processes for making those foods. Wheat *qu*, which provides liquefaction and saccharifying power, and affects the flavor quality, is a key ingredient in ancient alcoholic *huangjiu* fermentation, while core microbiota of them still remains indistinct. In this study, metagenomics, metabolomics, microbial isolation and co-fermentation were used to investigate *huangjiu*. Although *Aspergillus* is usually regarded as core microorganism in wheat *qu* to initiate *huangjiu* fermentations, our metagenomic analysis showed that bacteria *Saccharopolyspora* are predominant in wheat *qu* and responsible for breakdown of starch and cellulose. Metabolic network and correlation analysis showed that *Saccharopolyspora rectivirgula, Saccharopolyspora erythraea*, and *Saccharopolyspora hirsuta* made the greatest contributions to *huangjiu*’s metabolites, consisting of alcohols (phenylethanol, isoamylol and isobutanol), esters, amino acids (Pro, Arg, Glu and Ala) and organic acids (lactate, tartrate, acetate and citrate). *S. hirsuta* J2 isolated from wheat *qu* had the highest amylase, glucoamylase and protease activities. Co-fermentations of *S. hirsuta* J2 with *S. cerevisiae* HJ resulted in a higher fermentation rate and alcohol content, and *huangjiu* flavors were more similar to that of traditional *huangjiu* compared to co-fermentations of *Aspergillus* or *Lactiplantibacillus* with *S. cerevisiae* HJ. Genome of *S. hirsuta* J2 contained genes encoding biogenic amine degradation enzymes. By *S. hirsuta* J2 inoculation, biogenic amine content was reduced by 45%, 43% and 62% in *huangjiu*, sausage and soy sauce, respectively. These findings show the utility of *Saccharopolyspora* as a key functional organism in fermented food products.

## Introduction

For millennia, humans have continuously optimized the growth and metabolic conditions of specific microbial communities. This is best exemplified for food fermentations, an ancient food production method which through repeated production practices resulted in the repetitive enrichment and eventual domestication of certain microorganisms responsible for defining flavor and quality characteristics^1–3^. However, the core microorganisms required for producing many traditional fermented foods and their specific contributions to the final food products remain to be determined.

**Fig. 1:**
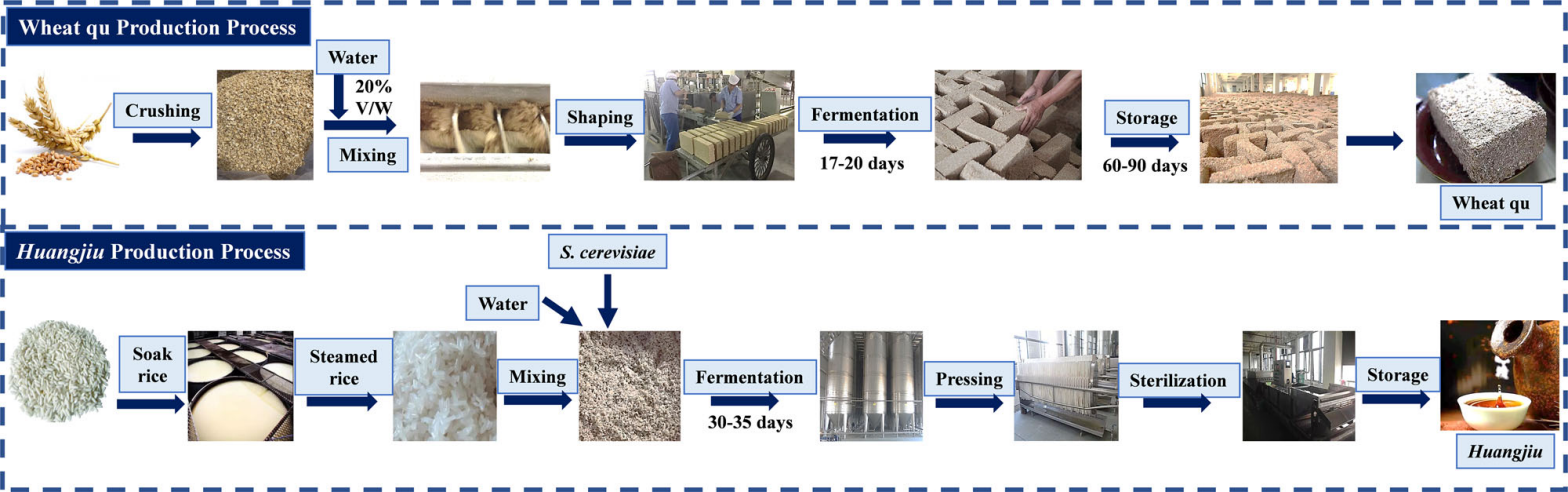
Manufacture chart of wheat *qu* and HJFM. Wheat *qu* Production Process: (I) Crushing, Mixing and Shaping. The wheat is crushed, blended with water (~20% V/W), and then pressed into molds of different size depending on the manufacture, which are called wheat *qu* blocks. (II) Fermentation. the wheat *qu* blocks are moved into *qu* house for culturing under ventilated and insulated conditions. Fermentation is divided into three fermentation stages: First, 30 to 40 °C for 3 days, which results in the mass reproduction and growth of microorganisms. Second, 45 to 55 °C for 4 to 5 days, which is used mainly for the accumulation of microbial metabolites. Third, 10 to 12 days above 40 °C, which is conducive to moisture removal in the starter core and the formation of aroma substances. The wheat *qu* were dried to a water content below 12% weight. (III) Storage. After cooling downing to room temperature, the wheat *qu* was stored for 60 to 90 days. *Huangjiu* Production Process: (I) Soak rice. The rice is washed and soaked in natural water at room temperature for 40 h. (II) Steamed rice. The soaked rice is steam-cooked in a steaming machine to gelatinize the starch contained in the rice grains. (III) Mixing. The steamed rice is mixed with wheat *qu*, water and *S. cerevisiae* at a weight ratio of 15:1.4:12.5:1.1, and the mixture is called *huangjiu* fermentation mash (HJFM). (IV) This process can be divided into two stages. The stage I fermentation temperature is 28–32 °C and lasts for 3–4 day, and stage II lasts 15 to 20 days and the temperature controlled below 15 °C. (IV) Pressing, sterilization and storage. After fermentation, the HJFM were filtered by pressing. Sterilize and store the fresh *huangjiu*.

*Huangjiu* is one of China’s most popular alcoholic beverages and archeological records suggest this beverage has been made for nearly nine millennia^4,5^. *Huangjiu* is made as a result of a complex, spontaneous, mixed-culture process in an open-air environment. There are two main methods for *huangjiu* fermentation, traditional method and industrial method. The principles of these two methods are similar, except for producing time and some materials. In traditional method, wheat *qu* is prepared in summer, materials are prepared in winter, and the overall fermentation needs almost one year, while in industry materials can be prepared in anytime and the fermentation is much faster. Meanwhile, comparing to traditional fermentation, industrial fermentation needs additional *Aspergillus flavus* SU16. The industrial *huangjiu* production process usually involves several processes wherein the rice is soaked, steamed, and a two-stage *huangjiu* fermentation process (Fig. 1). *Saccharomyces cerevisiae*, wheat *qu*, steamed glutinous rice, and water were the major ingredients for h*uangjiu* fermentation, and the mixture is called *huangjiu* fermentation mash (HJFM). The wheat *qu* starter culture which is made by wheat fermentation affects the flavor quality of the final product and contains *Aspergillus, Rhizopus*, and other fungi^6,7^. The primary contribution of *S. cerevisiae* is ethanol production^8^, while the primary contribution of wheat *qu* is to provide liquefaction and saccharifying power, which also affects the flavor quality of the final product. Although wheat *qu* is thus a key ingredient in ancient alcoholic *huangjiu* fermentation, the core microbiota of them still remains indistinct. Additionally, *Aspergillus flavus*, a filamentous fungus, has been widely used in large-scale production of traditional fermented food products, such as sake, miso, soy sauce, and *huangjiu*^9,10^. *A. flavus* SU-16 (also known as *Aspergillus oryzae* SU16) is often inoculated into cooked wheat *qu* and used as a supplement of enzyme preparation in *huangjiu* fermentation^9^. Although *A. flavus* SU-16 have been used for industrial wheat *qu* preparation^11^, the aroma characteristics of resulting *huangjiu* are significantly different from traditional *huangjiu*^5^. Therefore, there remains the need to identify the core microbiota of wheat *qu* and *huangjiu* responsible for the characteristics of the traditional *huangjiu* beverage.

Flavor compounds are very important indicators of *huangjiu’s* quality^12,13^. Currently, more than 900 kinds of volatile flavor compounds have been detected in *huangjiu*, including acids, alcohols, aldehydes, esters, ketones, and phenols. The flavor compounds are greatly affected by microorganisms in *huangjiu* brewing, while the formation mechanism is still poorly understood. Besides its unique flavor compounds, *huangjiu* is also known to contain biogenic amines (BAs)^14^. These compounds are low molecular weight, non-volatile nitrogenous organic bases made from the decarboxylation of amino acids^15^. BAs contribute to the normal physiological function of the human body, however, high intake may also result in the adverse physiological reactions of headache, nausea and other allergic reactions^3,12^. The rice soaking and *huangjiu* fermentation process result in increasing concentrations of the BAs, including histamine, tyramine, putrescine and cadaverine^16^. Although the quantities of BAs in *huangjiu* (39.30–241 mg/L) are reportedly below levels known to cause adverse reactions^14,17^, recent studies have shown that BAs are responsible for the unpleasant “hangover” symptoms after drinking *huangjiu*^12^. Reducing the BAs concentration is a viable way to improve the quality and taste of *huangjiu*, and other fermented food products that contain high concentration of BAs, such as sausage and soy sauce.

Accordingly, in this study, a comprehensive systems biology approach was applied using metagenome sequencing, metabolomics, metabolic pathways, and metabolites analysis to identify the core functional microorganisms in wheat *qu* and *huangjiu* fermentations. This approach resulted in the discovery of *Saccharopolyspora* as a key member of the *huangjiu* microbiome and the role of members of this genus in BAs metabolism. Meanwhile, microbial strains that were involved in degradation of BAs in wheat *qu* and *huangjiu* fermentation was isolated, and its potentials and metabolic activities to reduce BAs in *huangjiu* were evaluated by co-culture fermentation for the first time, as well as other fermentations with high concentration of BAs, such as sausage and soy sauce. The analysis showed us in-depth views of core functional microorganisms in *huangjiu* fermentations, and provided a viable way to reduce the BAs in *huangjiu* and other fermented food.

## Results

### *Saccharopolyspora* is abundant in the wheat qu and HJFM microbiota

**Fig. 2:**
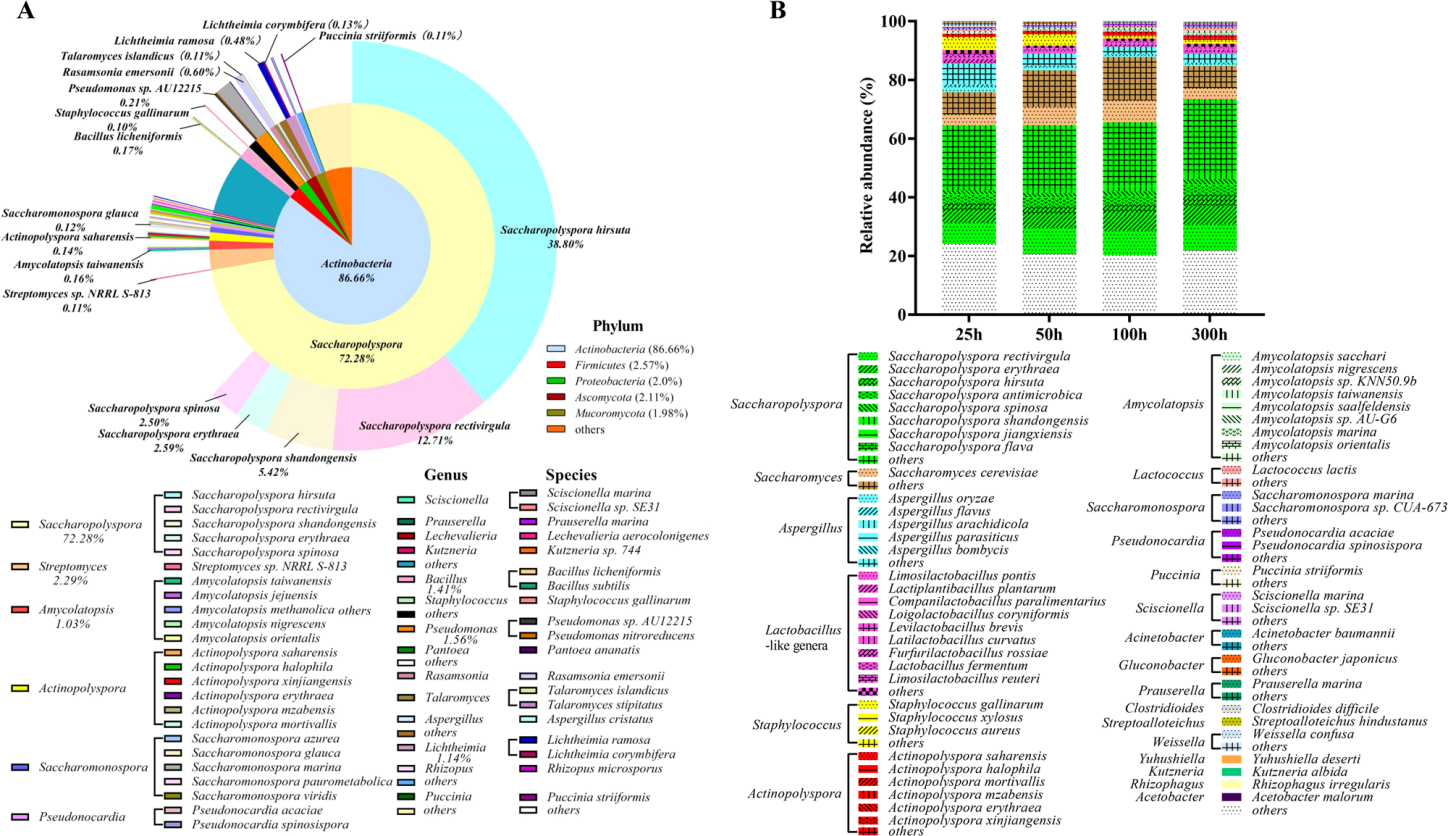
Microbial communities in wheat *qu* and *huangjiu* fermentation. **A** Microbial communities of wheat *qu* at phylum (inner circles), genus (intermediate circle) and species levels (outer circles). **B** Dynamics of functional microorganism during *huangjiu* fermentation process at species level.

To uncover the variation curve of microorganisms during fermentation, microbial DNA copy numbers and community structure were analyzed using quantitative real-time PCR (qPCR) and metagenomic analyses. DNA copy numbers of total bacteria and fungi in wheat *qu* were 11.2 ± 0.2 log10 copies/g and 6.6 ± 0.1 log10 copies/g. Metagenome sequencing showed a total of 430 genera (including 263 bacteria and 167 fungi) and 1442 (including 1176 bacteria and 266 fungi) species in the wheat *qu* sample obtained from a commercial *huangjiu* factory (Supplementary Table 1). Bacteria were dominant, constituting 90.24% of the microorganisms present and were predominantly in the *Actinobacteria* (86.66%) phylum (Fig. 2A). Only six genera were detected at >1% abundance, and *Saccharopolyspora* (72.28%), and *Streptomyces* (2.29%) were the most represented. Other taxa included *Bacillus* (1.41%), *Pseudomonas* (1.56%), *Amycolatopsis* (1.03%) and *Lichtheimia* (1.14%). Metagenome sequences were also able to resolve species-level differences. *S. hirsuta* was the most abundant (38.80%), followed by other species of *Saccharopolyspora*, including *S. rectivirgula* (12.71%), *S. shandongensis* (5.42%), *S. erythraea* (2.59%), and *S. spinosa* (2.50%).

DNA copy numbers of total bacteria and total fungi in HJFM rapidly increased to 6.70 ± 0.1 log copies/g and 5.81 ± 0.0 log copies/g in the first 25 h of fermentation, and reached 12.55 ± 0.3 and 8.90 ± 0.3 log copies/g at 50 h, respectively. Bacterial DNA copy number was maintained at 12.55 to 11.72 log copies/g between 50 and 100 h of fermentation, while fungal DNA copy number gradually increased from 8.90 to 10.77 log copies/g between 50 and 100 h of fermentation. At 300 h, bacterial and fungal DNA copy numbers decreased to 8.60 ± 0.3 log copies/g and 7.34 ± 0.1 log copies/g, respectively. At the 25, 50, 100 and 300 h time points during the fermentation, the microbial contents of the HJFM were mainly comprised of *Actinobacteria, Firmicutes*, and *Ascomycota* phyla (Supplementary Table 2). The relative abundance of the dominant genus, *Saccharopolyspora*, increased from 40.37% to 51.48% during *huangjiu* fermentation process (Fig. 2B and Supplementary Table 2). A variety of *Saccharopolyspora* species were detected including *S. rectivirgula, S. erythraea, S. hirsuta, S. antimicrobica, S. spinosa*, and *S. shandongensis* (Fig. 2B and Supplementary Table 2). *Saccharomyces*, specifically *S. cerevisiae*, was also present in the HJFM, and reached the highest levels (22.28%) in the HJFM 100 h post-incubation and then declined to 11.34%. *Aspergillus* was also present in more modest amounts (9.82%) after 25 h incubation, but declined over time to 4.17% of the total microbial contents by 300 h.

### Metabolites profiling of wheat *qu* and HJFM

The abundant metabolites were the most outstanding feature of Huangjiu, comparing to other fermentation fermented alcoholic beverage. In this study, A total of 103 metabolites were identified in the wheat *qu* sample tested, including 8 organic acids, 18 amino acids, and 77 volatile compounds. Tartrate (2.99 g/kg wheat *qu*), lactate (2.65 mg/kg wheat *qu*) and acetate (1.91 mg/kg wheat *qu*) accounted for 81.53% (9.26 mg/kg wheat *qu*) of the 8 organic acids detected (Supplementary Fig. 1A). Pro, Glu, Arg, Asp, Ala and Lys were the most abundant amino acids in the wheat *qu*, comprising 70.14% (744.48 mg/kg wheat *qu*) of the 18 amino acid quantities detected (Supplementary Fig. 1B). The total content of the volatile compounds in wheat *qu* were found to be 1762.37 μg/kg wheat *qu*. Among the identified volatile compounds, alcohols (phenylethanol, 1-octen-3-ol, 2,3-butanediol, 2-octene-1-ol) and esters (hexanoate ethyl, ethyl palmitate, ethyl acetate, dibutyl phthalate) were the most abundant (relative abundances of 65.7% and 14.5%, respectively).

Metabolites present in the HJFM changed over time (Supplementary Fig. 1). Lactate, tartrate, and citrate were the dominant organic acids in HJFM, comprising between 86.91% to 90.00% of the 8 organic acids identified (Supplementary Fig. 1A). Seven amino acids (Arg, Try, Ala, Pro, Glu, Leu and Gly) accounted for between 58.42% (400 h) to 70.19% (100 h) of the 18 amino acids detected (Supplementary Fig. 1B). 64 volatile compounds were also identified in the HJFM, 5 alcohols (phenylethanol, isoamylol, isobutanol, 3-methylthiopropanol, and 2,3-butanediol), 10 esters (ethyl lactate, ethyl acetate, diethyl succinate, ethyl butyrate, isoamyl acetate, isobutyl acetate, phenethyl acetate, ethyl decanoate, ethyl octanoate, and ethyl palmitate) and 3 phenols (4-vinylguaiacol, 4-ethyl guaiacol, and 4-ethyl phenol) were the main volatile compounds of HJFM, comprised 89.28% to 99.49% of the total content (Supplementary Fig. 1C). Among the identified volatile compounds, the aroma compounds phenylethanol, isoamylol, isobutanol, 3-methylthiopropanol, ethyl lactate, ethyl acetate, 4-vinylguaiacol, and 4-Ethyl guaiacol were the most abundant (relative abundances from 87.16% to 97.47%) in HJFM.

### Identification of core functional microorganisms in HJFM microbiota by metabolic pathways analysis

To identity the core functional microorganisms, metabolic pathway analysis was performed. The HJFM metagenomes confirmed that *Saccharopolyspora* was likely the core functional microorganism in *huangjiu* fermentation (Supplementary Fig. 2). Pathway analysis (Supplementary Fig. 2A-a) showed that the enzymes (EC 3.2.1.4 and 3.2.1.91) responsible for cellulose metabolism were highly enriched in *Saccharopolyspora* (43.09%) and *Amycolatopsis* (42.78%). *Aspergillus* (14.03%) was also associated with that function in the metagenomes. Many microorganisms, including *Saccharopolyspora* (71.05%) and *Saccharomyces* (19.20%) had the capacity to participate in extracellular starch degradation (Supplementary Fig. 2B-a). The grain proteins of raw material can be hydrolyzed into amino acids or peptides by the action of enzymes (EC 3.4.--, 3.4.11.1, 3.4.11.2, 3.4.13.-, 3.4.16.4, and 3.4.17.14) in HJFM metagenomes, and 10 microorganisms may be involved in protein degradation, among which, *Saccharopolyspora* (82.88%) might be the main actors.

Phenylethanol, isoamylol and isobutanol are the most abundant higher alcohols in wheat *qu* and HJFM. In both catabolic (Ehrlich pathway) and biosynthetic pathways, α-ketoic acids are converted into higher alcohols by carboxylases (EC 4.1.1.-) and dehydrogenases (EC 1.1.1.1, 1.1.1.2, and 1.1.1.90) (Supplementary Fig. 2A) in HJFM microbiota. *Saccharomyce*s (36.48%), *Saccharopolyspora* (33.33%), *Actinopolyspora* (8.63%), and *Amycolatopsis* (5.87%) may also be involved in higher alcohol synthesis (Supplementary Fig. 2B-c).

Ethyl esters and acetic esters, which are mostly fruity and floral, are important esters in HJFM. Lipases (EC 3.1.1.1, EC 3.1.1.3), the key ester biosynthetic enzymes during fermentation, can catalyze the reversible condensation of acid and alcohols in this study (Supplementary Fig. 2A). As shown in Supplementary Fig. 2B-b, many microorganisms, including *Saccharomyces* (59.75%), *Saccharopolyspora* (25.23%) and *Staphylococcus* (10.66%) had the capacity to synthesize esters. Among them, *Saccharomyces* and *Saccharopolyspora* might provide the greatest ester biosynthetic capacity.

Pro, Glu, Arg and Ala were the main amino acids in wheat *qu* and HJFM, and a number of genes that may participate in amino acid metabolism were observed in the study. Ornithine cyclodeaminase (EC 4.3.1.12) can transform ornithine to Pro, while pyrroline-5-carboxylate reductase (EC 1.5.1.2) can catalytic synthesis of Pro with L-glutamate-5-semialdehyde as the precursors (Supplementary Fig. 2A). Arg can be synthesized from citrulline or l-argininosuccinate by enzymes (EC 3.5.3.6, 1.14.13.39, 1.14.14.47, and 4.3.2.1). Transaminases (EC 2.6.1.1, EC 2.6.1.2) and dehydrogenases (EC 1.4.1.2, 1.4.1.3, and 1.4.1.4) can catalyze the formation of Glu from 2-oxoglutarate. Glu can also be synthesized from l-glutamate-5-semialdehyde using L-glutamate gamma-semialdehyde dehydrogenase (EC 1.2.1.88). Ala is synthesized from pyruvate in a reaction catalyzed by transaminases (EC 2.6.1.2, 2.6.1.21) and alanine dehydrogenase (EC 1.4.1.1). Besides, Asp can be converted into Ala by aspartate 4-decarboxylase (EC 4.1.1.12). *Saccharopolyspora* (69.92%) and *Saccharomyces* (20.20%) may also be involved in amino acids synthesis (Supplementary Fig. 2B-d).

Lactate, tartrate, acetate and citrate were the main organic acids in wheat *qu* and HJFM. Based on the result of metagenome annotation, pyruvate (related enzymes EC 1.1.99.40, 1.1.1.27, 1.1.1.28, 1.1.5.12) is the immediate precursors of lactate synthesis (Supplementary Fig. 2A). It was determined that those related enzymes genes were encoded by *Saccharopolyspora* (58.90%)*, Saccharomyces* (18.23%), and *Staphylococcus* (5.06%) (Supplementary Fig. 2B-e). *Saccharopolyspora* (80.37%) have the capacity to transform oxaloacetate into tartrate using tartrate dehydrogenase/decarboxylases (ttuc, EC:1.1.1.93, 4.1.1.73, and 1.1.1.83). The acetate formation pathways vary across different microbial genera, and *Saccharopolyspora* (76.63%) and *Saccharomyces* (13.66%) are the main acetate producers. Citrate is a well-known intermediate in the TCA cycle. *Saccharopolyspora* (54.13%) and *Saccharomyces* (38.42%) may participate in the formation of citric acid in HJFM.

In summary, *Saccharopolyspora* should be a major player in raw material (cellulose, starch and protein) degradation and the synthesis of all the main metabolites. A total of 122 *Saccharopolyspora* genes encoding flavor-forming enzyme were identified, and most of the genes were present at high abundance. *Saccharomyces* may participate in starch degradation, and the synthesis of esters, higher alcohols, amino acids, lactate, acetate and citrate. *Aspergillus* may be involved in cellulose degradation.

### O2PLS-based statistical correlations between core functional microorganisms and flavor metabolites

**Fig. 3:**
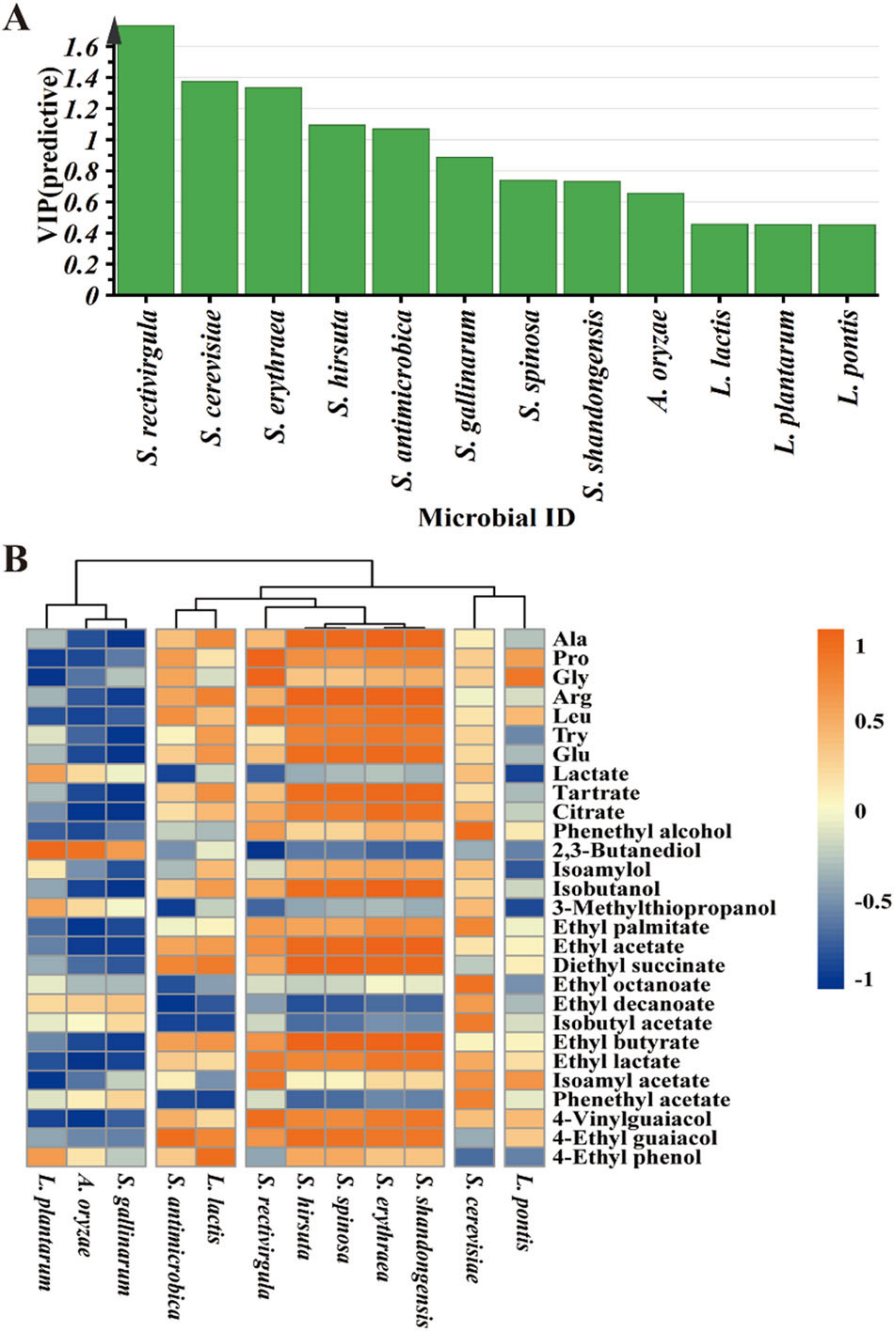
Correlation analyses between core functional microorganisms and main metabolites by O2PLS modeling during the HJFM process. **A** *VIP*_(*pred*)_ (variable importance for predictive components) plot of the core functional microorganisms (only those with relative abundance ≥1%) (**B**) Pheatmap of the main metabolites and the core functional microorganisms during the HJFM process. The correlation coefficients are represented by the color. Red indicates positive correlation and blue indicates negative correlation. Correlations were calculated based on relative abundance and absolute quantification. Clustering analysis was performed using the Pearson correlation coefficient and Euclidean distance.

To explore the correlation between the microbiota and main metabolites, a bidirectional orthogonal partial least squares (O2PLS) method was used to analyze the change in the relative abundance of core functional microorganisms (only those with relative abundance ≥1%) and main flavor metabolites (total 7 amino acids, 3 organic acids and 18 volatile compounds) during the fermentation of HJFM over time to explore the correlation between the microbiota and main flavor metabolites (Supplementary Table 3). According to the model, the proportions of five microbial species (*VIP*_*(pred)*_ > 1.0) (including four bacterial (*S. rectivirgula, S. erythraea, S. hirsuta* and *S. antimicrobica*) and one fungal (*S. cerevisiae*) species) had a significant influence on the flavor metabolites (Fig. 3A). Among those taxa, the O2PLS model showed that *Saccharopolyspora* was the most important contributor to the production of flavor metabolites during *huangjiu* fermentation. Based on the correlation coefficient between microbiota and flavor metabolites (Fig. 3B), S. *rectivirgula, S. erythraea, S. hirsuta, S. antimicrobica* and *S. cerevisiae* was positively correlated with 6, 16, 14, 2 and 5 of the main flavor metabolites, respectively (*ρ* > 0.7). Among them, phenethyl alcohol, isobutyl acetate, ethyl octanoate and phenethyl acetate were only associated with *S. cerevisiae* proportions (*ρ* > 0.7). This result indicated that both *S. erythraea* and *S. hirsuta* shared a positive correlation with five amino acids (Ala, Arg, Leu, Try, and Glu), two organic acids (tartrate and citrate), and six volatiles (ethyl acetate, diethyl succinate, ethyl butyrate, ethyl lactate, 4-vinylguaiacol, and 4-ethyl guaiacol). The proportions of *S. rectivirgula* were positively correlated with six flavor metabolites (*ρ* > 0.7, Pro, Gly, Leu, ethyl lactate, isoamyl acetate, and 4-vinylguaiacol), while *S. antimicrobica* was only positively correlated with diethyl succinate and 4-ethyl guaiacol. In addition, the proportions of *Lactiplantibacillus plantarum*, *Aspergillus oryzae*, *S. gallinarum* were strongly negatively correlated with 7, 13, and 13 main flavor metabolites, respectively, indicating a potential role in flavor metabolite degradation. In conclusion, based on this model with this single HJFM measured over time, *S. erythraea*, *S. hirsuta, S. rectivirgula* and *S. cerevisiae* may be the core metabolites producing microorganisms in *huangjiu* fermentations.

### Enzymatic activities of *Saccharopolyspora* isolated from wheat qu

To better understand the role of *Saccharopolyspora* in *huangjiu* fermentations, we isolated bacteria from the wheat *qu*. Isolated strains were identified at the species level by 16 S rRNA gene sequencing and found to be *S. hirsuta* (J1 to J3), *S. rectivirgula* (J4 and J5), *S. shandongensis* (J6 to J8) and *S. erythraea* (J9). Among the isolates, *S. hirsuta* J2 showed the highest activity of amylase, glucoamylase and protease (Supplementary Fig. 3). The protease and glucoamylase activities of *S. hirsuta* J2 were 2927.74 ± 16.13 U/g and 2.41 ± 0.06 U/g, respectively, which were significantly higher than those in the control group (*P* < 0.05), but there were no significant differences in amylase activity (*P* < 0.05). The amylase, glucoamylase and protease activities of *A. flavus* SU-16 were significantly higher than control group (*P* < 0.05). The glucoamylase and protease activities of *A. oryzae* MQ were significantly higher than control group (*P* < 0.05), while there was no significant difference in protease activity. And the three enzyme activities *of L. plantarum* JN01 were significantly lower (*P* < 0.05).

### *Huangjiu* made with *S. hirsuta* J2 has improved metabolites and fermentation characteristics

**Fig. 4:**
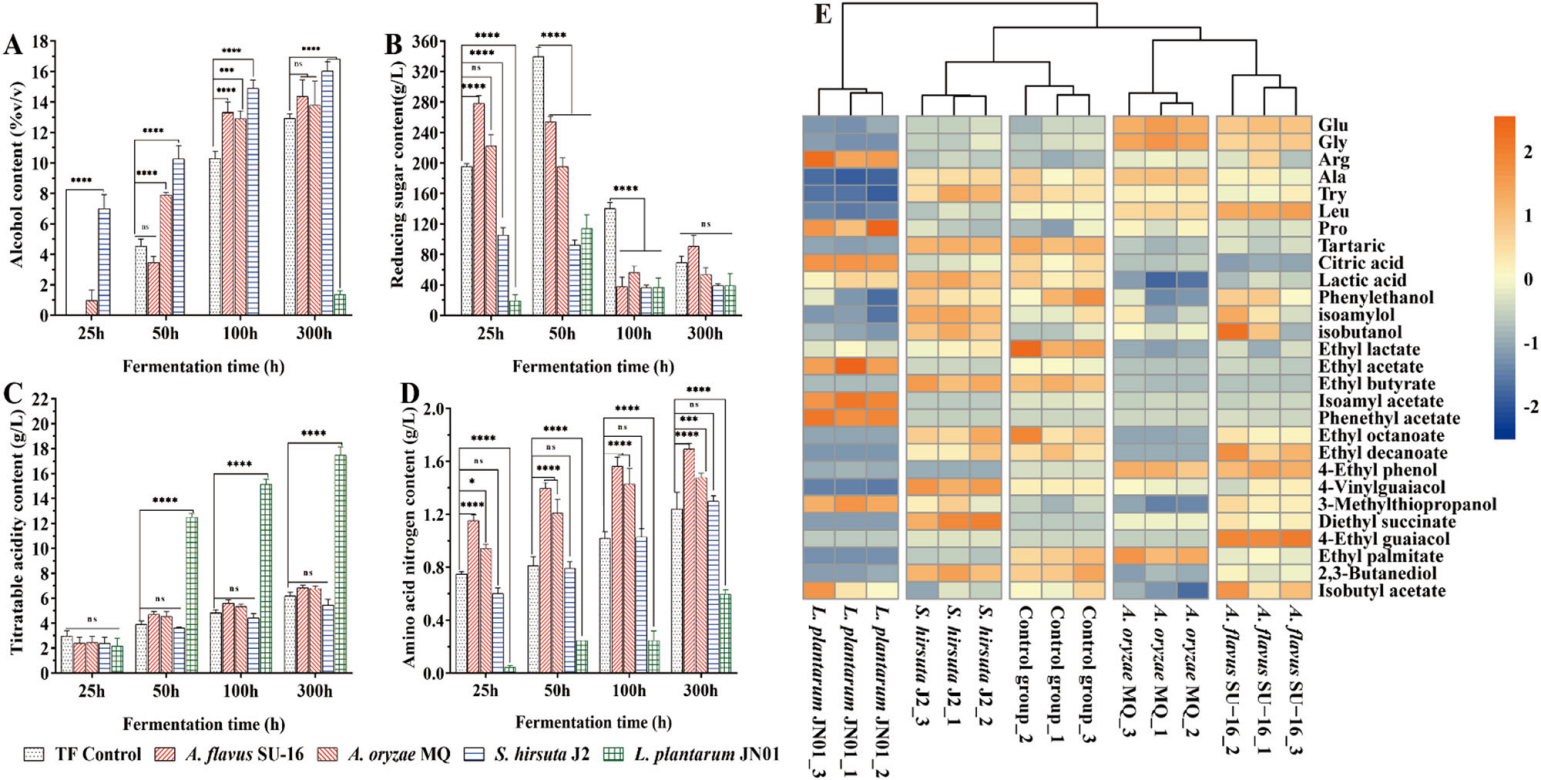
Physicochemical parameters and main metabolites during the HJFM process. **A** Alcohol content (g/L); (**B**) Reducing sugar content (g/L); (**C**) Titratable acidity content (g/L); (**D**) Amino acid nitrogen content (g/L); (**E**) Heatmap of the main metabolites during the HJFM process. Clustering analysis was performed using the Pearson correlation coefficient and Euclidean distance based on the main flavor metabolites contents during the fermentation process. A value of 0.05 was set as the significance level; the data were marked as (*) *p* < 0.05, (**) *p* < 0.01, (***) *p* < 0.001, and (****) *p* < 0.0001. The *p*-value above 0.05 was considered as non-significant (ns). Error bars in histogram mead the standard deviation of observed data.

Because of its highest activity of amylase, glucoamylase and protease, *S. hirsuta* J2 was selected for the subsequent fermentation experiment. Lab-scale (3 L) *huangjiu* fermentations were prepared with *S. cerevisiae* HJ and either *S. hirsuta* J2, *A. flavus* SU-16 (used in *huangjiu* production), *A. oryzae* MQ (used in sake production), or *L. plantarum* JN01 (used in sake and sherry production). Figure 4 shows the main physicochemical results of the *huangjiu* according to the national standards of China (GB/T 13662-2018). All the final fermentation products met the national standards except for *huangjiu* inoculated with *L. plantarum* JN01, Fermentations with *S. hirsuta* J2 had the highest fermentation rate (0 to 7.00% v/v in 25 h) and final alcohol content (16.03% v/v at 300 h) than fermentations inoculated with the other strains or the control group (traditional fermentation group) (*P* < 0.01) (Fig. 4A). Similarly, the reducing sugar content of the *S. hirsuta* J2 group remained low (<120 g/L) during the fermentation, and was significantly lower than that of the control group and those containing *Aspergillus* (Fig. 4B). Compared with the control group, the speed of *A. flavus* SU-16 and *A. oryzae* MQ groups fermentations were slightly higher, however the final alcohol contents (300 h) were no significant difference with the control group. At 300 h, sugar in *A. flavus* SU-16 and *A. oryzae* MQ groups was exhausted, and their values were not significantly different from these of the control groups. The fermentation inoculated with *L. plantarum* JN01 did not result in ethanol production (<2% v/v), but the reducing sugar content increased first and then decreased as the fermentation proceeds, and the maximum value is 114.06 ± 18.02 g/L at 50 h. The titratable acid content of the *L. plantarum* JN01 group also increased rapidly to 17.50 g/L, and the samples showed obvious rancidity (Fig. 4C). The titratable acid content of the other groups increased with fermentation, but remained within a reasonable and stable range. By the end of fermentation, the amino nitrogen content of each sample was in the 0.60–1.69 g/L range (Fig. 4D), which meets the superior standard of *huangjiu*^18^.

The metabolites of *huangjiu* fermented with *S. hirsuta* J2, *A. flavus* SU-16, *A. oryzae* MQ, and *L. plantarum* JN01 and the control group were determined, and 28 main metabolites were identified. The different metabolites of *huangjiu* fermented with *S. hirsuta* J2 had similar proportions compared to those in the control group (Fig. 4E). Hierarchical cluster analysis showed similarities between *S. hirsuta* J2 and the control group based on their main metabolites. These results indicate that the metabolite profile of traditional *huangjiu* could be simulated by co-fermentation of *S. hirsuta* J2 and *S. cerevisiae*. By comparison, concentrations of metabolites were similar between *huangjiu* containing the *A. flavus* SU-16 and *A. oryzae* MQ inoculants, but not to the controls.

### *S. hirsuta* J2 has the capacity to degrade BAs

**Fig. 5:**
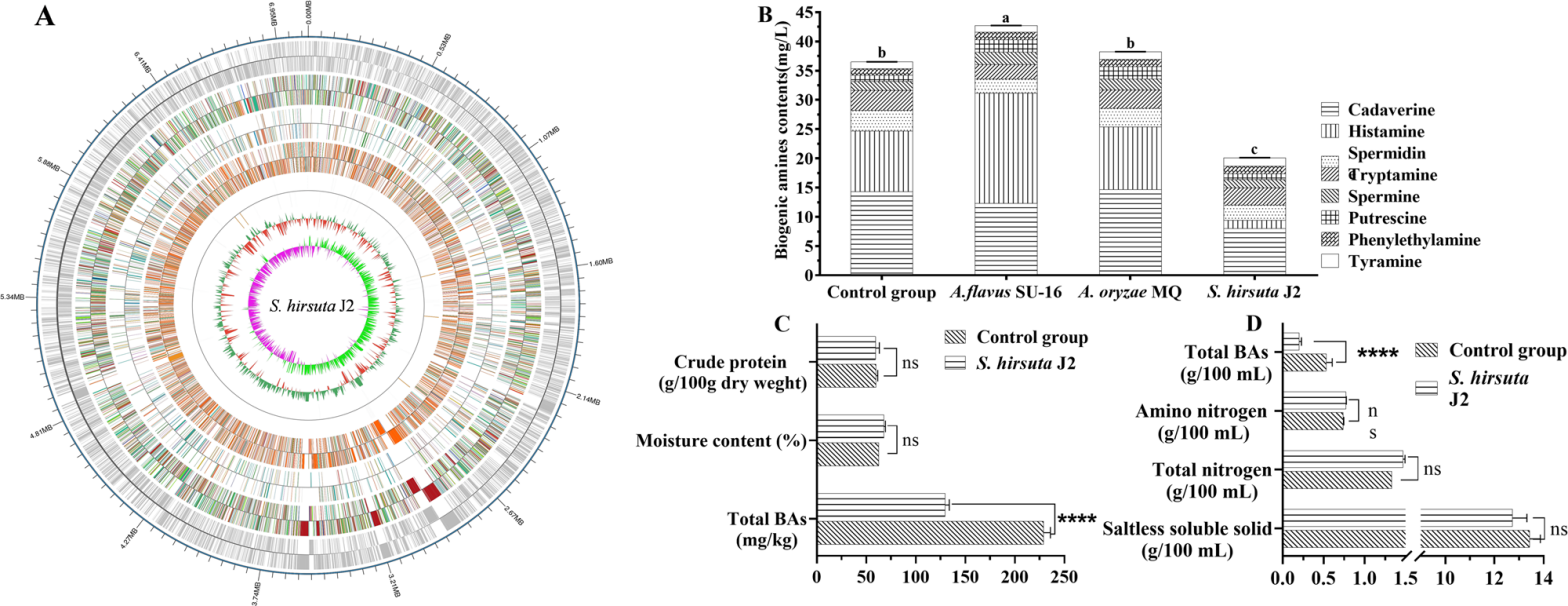
Genome and fermentation features of *S. hirsuta* J2. Circular maps of S. *hirsuta* J2 genomes (**A**). From the outer circle to the inner circle: Circle 1, the location coordinates of the genome sequence of *S. hirsuta* J2; Circle 2, the GC content (71.42%); Circle 3, the GC skew value. Circle 4, annotation distribution of genome coding genes; Circle 5, annotation distribution of rRNA gene; Circle 6, annotation distribution of ncRNA gene; Circle 7, annotation distribution of tRNA gene; Circle 8, the genome size is 7.08 M. The BAs content and physicochemical parameters of *huangjiu* (**B**), sausage (**C**) and soy sauce (**D**) Error bars in histogram mead the standard deviation of observed data.

The *S. hirsuta* J2 genome (Fig. 5A) encodes genes required for BAs degradation, including primary-amine oxidase (EC 1.4.3.21), acetyltransferase (EC 2.3.1.-) and spermidine synthase (EC 2.5.1.16) (Supplementary Table 4), according to the KEGG pathway analysis. These three enzymes bave the ability to degrade tyramine, cadaverine, phenylethylamine, putrescine, and histamine based on KEGG pathway analysis. This result indicates that *S. hirsuta* J2 has the potential to degrade BAs. Therefore, BAs production and degradation abilities of the *S. hirsuta* J2 was examined (Supplementary Fig. 4). It was found that the total content of BAs produced by *S. hirsuta* J2 was less than 2 mg/L, indicating that the strain basically did not produce BAs. The degradation rate of histamine, phenylethylamine and tyramine in the medium were more than 98%. The degradation rate of total BAs by *S. hirsuta* J2 was 72.30 ± 1.25%.

### *S. hirsuta* J2 reduces BAs content in *huangjiu*, soy sauce, and sausage

Before the fermentation was started, content of total BAs was determined to be 6.11 ± 0.32 mg/L in the HJFM (without cooked wheat *qu*). After the fermentation, the concentration of total BAs in *huangjiu* inoculated with *S. hirsuta* J2 (20.05 ± 0.43 mg/L) was significantly lower than *huangjiu* inoculated with *A. flavus* SU-16, *A. oryzae* MQ, and the control group (*P* < 0.01) (Fig. 5B). Histamine concentrations were reduced from 10.38 mg/L to 1.40 mg/L in *huangjiu* made with *S. hirsuta* J2, constituting an 86.51% reduction compared to the control group. Cadaverine and spermidine were also reduced by approximated 43.96% and 25.42%, respectively. By comparison, total BAs concentrations increased in *huangjiu* inoculated with *A. flavus* SU-16 from 36.48 ± 1.08 mg/L to 42.71 ± 1.16 mg/L compared to the control group. BAs content when *A. oryzae* MQ was added was indistinguishable from the control group.

To further explore the capacity of *S. hirsuta* J2 to degrade BAs in fermented foods, *S. hirsuta* J2 was inoculated into sausages and soy sauce for fermentation (Fig. 5C, D). The pH, moisture content, crude protein content, and soluble solid, total nitrogen and amino nitrogen content in soy sauce and sausage made with *S. hirsuta* J2 were not significantly different from the controls (*P* > 0.05). At the end of fermentation, BA concentration in the soy sauce and sausage of control group was 0.53 ± 0.06 g/100 mL and 229.03 ± 12.07 mg/kg, respectively. These values were lower when *S. hirsuta* J2 was added to the ferment, constituting a significantly decreased total BAs 43.41 ± 4.59% and 61.77 ± 6.22% compared with the control group in sausage and soy sauce fermentation, respectively.

## Discussion

Fermented foods play a major role in the diets of many cultures worldwide. At present, fermented foods are increasingly recognized for their regional heritage^19^. Traditional fermented foods are usually produced by natural fermentation containing a multispecies community^20^. Generally, there are only limited number of known core species responsible for driving the fermentation process^21^. The transformation from natural fermentation to tractable fermentation with the core functional microorganism is essential for consistent quality of fermented foods. Although *huangjiu* can be made from other matrixes such as corn^22,23^, rice is the mostly used matrix for *huangjiu* fermentation, so the rice *huangjiu* is being studied here.

In this study, metagenome sequencing, metabolomics, strain isolation and characterization were combined to identify the core microbial taxa in *huangjiu* fermentations and the corresponding metabolites that they make. This was possible even though *huangjiu* is a highly complex food fermentations that has multiple fermentation steps and requires diverse bacterial and fungal species. Specifically, we showed that *Saccharopolyspora*, a genus of bacteria that has thus far only been associated with the production of bioactive natural products, is a key member of the *huangjiu* fermentation. The associations of *Saccharopolyspora* with metabolites was verified using *S. hirsuta* J2, a strain isolated from *huangjiu*. The utility of this strain was further confirmed by showing that it may be useful for reducing biogenic amines in *huangjiu* as well as other fermented food types.

*Saccharopolyspora* is the dominant member of the wheat *qu* and HJFM (*huangjiu*) microbiota. The genus *Saccharopolyspora* was first established by Lacey & Goodfellow^24^ and was assigned to the family *Pseudonocardiaceae*. Members of this genus are found in a wide range of habitats, including soil, marine sediments, marine invertebrates, plants and clinical samples. Secondary metabolites from *Saccharopolyspora* include the antibiotic erythromycin and the insecticides. Previous studies indicated that *Saccharopolyspora* was detected in vinegar^25^, nuruk^26^, daqu^27,28^, and *huangjiu*^29^. However, the importance of these bacteria in food fermentations was not determined. Based on O2PLS analysis, *S. rectivirgula, S. erythraea, S. hirsuta, S. antimicrobica* and *S. cerevisiae* were the primary contributors to the *huangjiu* metabolome. Among them, *S. erythraea* and *S. hirsuta* possessed the largest number of correlated metabolites (16 and 14 respectively), while *S. cerevisiae* was correlated with only 5 metabolites (*ρ* > 0.7, alcohols and esters). This indicated that *Saccharopolyspora* may be the main functional microorganism involved in the synthesis of metabolites in *huangjiu* fermentation, rather than *S. cerevisiae* or *Aspergillus*.

Our findings also showed that *huangjiu* fermentations (including wheat *qu* and HJFM) are dominated by bacteria, not fungi. This result is notable considering that *huangjiu* is an alcoholic fermentation, and *A. flavus* and *S. cerevisiae* is generally regarded as the main functional microorganisms in *huangjiu* fermentations^13,30,31^. *A. flavus* can produce a variety of extracellular enzymes such as α-amylase and glucoamylase that are responsible for the saccharification of rice. However, despite the known functional importance, *A. flavus* was of relative lower abundance, constituting only between 0.22% and 0.64% of the microbiota in HJFM. *S. cereviseae* is the traditional strain used to produce alcohol in the fermentation of alcoholic beverages^32^. In this study, as the fermentation progressed, the relative abundance of *S. cerevisiae* reaches up to 7.49% after the first stage (100 h), then decreased to 3.87% by stage II (300 h), which is consistent with its ethanol fermentation function. However, in addition to ethanol fermentation, *S. cerevisiae* from Shaoxing region was found to be mainly associated with the production of phenethyl alcohol and 3-methylthiopropanol^30^. Thus, the formation of the complex metabolites of *huangjiu* may originate from the metabolism of other microorganisms.

Based on this, we further explored the formation pathway of the main metabolites during *huangjiu* fermentation by mapping of the annotated genes identified in the metagenomes to the KEGG pathway. Our study found that *Saccharopolyspora*, *Saccharomyces*, and *Aspergillus* are the main participant in the synthesis of *huangjiu* metabolites. Moreover, correlation analysis suggested that *Saccharopolyspora* may be responsible for starch, cellulose and protein degradation during *huangjiu* fermentation, and not just the *Aspergillus* (like *A. flavus, A. oryzae*) as commonly thought^33^. Although *A. flavus* SU-16 has been used as the main resource of enzymes (protease, glucoamylase and liquifying enzyme etc.) in cooked wheat *qu* since 50 years ago. But additionally, *Aspergillus* may be involved only in the synthesis of Pro and Glu. *Saccharomyces* (*S. cerevisiae* and non-*Saccharomyces* yeast) may be involved in the synthesis of higher alcohols, esters, amino acids (Pro, Arg, Glu, Ala), and organic acids (acetate and citrate, lactate). Previous studies have found that non-*S*. yeast contribute to the production of esters and higher alcohols^34^. Also, in addition to being the main producer of lactate, it may be involved in the synthesis of higher alcohols.

Isolation and characterization of several *Saccharopolyspora* strains showed that these bacteria possess enzyme production (amylase, glucoamylase and protease) capacities. The utility of members of this genus was shown for *S. hirsuta* J2, an isolate from wheat *qu*. Inoculation of *S. hirsuta* J2 into HJFM could not only improve the rate of ethanol fermentation and final alcohol content, but also did not change the flavor of traditional *huangjiu*. There was no significant difference in fermentation parameters between the *S. hirsuta* J2 group and the control group (except alcohol content) at the end of fermentation. Importantly, the main metabolites of ferments containing *S. hirsuta* J2 were very similar to the control group. Additionally, the addition of *S. hirsuta* J2 resulted in a higher fermentation rate and alcohol content than control group (*P* < 0.0001). The increased fermentation rate may be due to improved breakdown of polysaccharides and proteins by amylases, glucoamylases, and acidic proteases produced by *S. hirsuta* J2 that result in more usable energy sources for *S. cerevisiae*.

BAs are a potential health risk substance and have been widely associated with food quality and safety^3,35^. In *huangjiu*, BAs are mainly produced in rice soaking and fermentation process^16^. There are no accurate regulations for BAs, but several countries including Germany, Belgium, France, and Switzerland have set regulations and limits for the upper limit of histamine, which are 2 mg/L, 6 mg/L, 8 mg/L and 10 mg/L respectively^36^. European Food Safety Authority (EFSA) recommended that the starter cultures lacking the capacity to produce BAs should be used when making fermented foods^37^. This is especially important in fermented food where high microbial activities may result in undesirable BAs accumulation^38^. Notably, *S. hirsuta* J2 can also degrade BAs. The total degradation rate of eight BAs was 72.30% in the culture medium, especially the six major BAs of tyramine (77.43%), putrescine (58.12%), histamine (99.55%), cadaverine (46.91%), phenylethylamine (98.19%) and tryptamine (98.41%). Although the capacity of *S. hirsuta* J2 to degrade BAs decreased slightly when added to complex fermentation environments, we found that compared with the control group, *S. hirsuta* J2 still degraded 45.05%, 43.41% and 61.77% of BAs content in *huangjiu*, soy sauce, and sausage. Notably, a different study showed that *L. plantarum* CAU 3823 isolated from *huangjiu* was able to degrade the BAs (40%)^39^. However, this value is slightly lower than *S. hirsuta*, suggesting the *S. hirsuta* J2 can adapt to different fermentation environments and can degrade BAs significantly.

By identifying the core functional organisms required to make fermented foods it may be possible to improve the scale and consistency of those product over time. Understanding the roles of microbial groups with important functions in various food fermentation systems has largely relied on bringing organisms into culture and assessing their metabolic capabilities. In recent years, metagenomics sequencing has been successfully used as an approach to connect taxonomy with these key fermentative functions and to assess the diversity of functional taxa in their fermentation systems^40–42^. *Saccharopolyspora* may be the potential functional strains leading the *huangjiu* fermentation. More studies on the application and function of *Saccharopolyspora* in food fermentation are required.

## Methods

### Wheat *qu* and HJFM sampling and pretreatment

Wheat *qu* and *huangjiu* fermentation mash were provided by a *huangjiu* factory (30°08′N, 120°49′E) in Shaoxing, Zhejiang, China on October 2018. As displayed in Fig. 1, a two-stage *huangjiu* fermentation process was performed in factory. The wheat *qu* was transported to the laboratory on dry ice within 24 h of collection and stored at −80 ^o^C until analysis. In order to obtain adequate information and representation before carrying out analysis, 12 wheat *qu* blocks were selected randomly from upper, middle and lower locations in the storage room, all the blocks were ground to powder in a sterile grinder and mixed as a sample. The size of each block of wheat *qu* is approximately 23 × 15 × 7.5 cm (length × width × high), weighing around 2.5 kg. The HJFM was sampled in 1000 g quantities after 25, 50, 100 and 300 h of the production process in 30-ton fermentation tanks. Prior to sampling, the HJFM was mixed by aeration machines to improve sample homogeneity. Samples were transported on dry ice to the laboratory and immediately stored at −80 °C for metabolite and DNA analysis.

### DNA isolation, quantitative real-time PCR (qPCR) and metagenome sequencing

Wheat *qu* and HJFM (5 g) were homogenized and grounded in liquid nitrogen, suspended in 10 mL of phosphate buffered saline buffer (PBS, pH 7.0), and then shaken for 5 min using a vortex adaptor. DNA was then extracted using sodium dodecyl sulfate and cetyl trimethyl ammonium bromide (SDS-CTAB)^11^. DNA concentrations were quantified using a UV-Vis Spectrophotometer Q5000 (Quawell, San Jose, USA). The DNA was stored at −20 °C until analysis. qPCR was used to detect the biomass of bacteria and fungi in wheat *qu* and HJFM according to the previous methods^43^. qPCR was performed using an qTOWER 3.0 system (Analytik Jena, Jena, Germany).

DNA libraries were constructed using 1 μg genomic DNA and the NEBNext® Ultra™ DNA Library Prep Kit (NEB, USA) following the manufacturer’s recommendations. Briefly, DNA was fragmented by sonication to a size of 350 bp, and then the DNA fragments were end-polished, A-tailed, and PCR was used to ligate a full-length adaptor for Illumina sequencing. The libraries were purified (AMPure XP system), evaluated for size distribution on an Agilent 2100 Bioanalyzer (Agilent Technologies), and quantified using real-time PCR according to the qPCR Quantification Protocol Guide (Agilent Technologies). The Illumina platform (Illumina Hiseq 4000) with 2 × 150 bp paired-end sequencing was used for sequencing each DNA library (Novogene, China).

### Metabolites profiling

To investigate relationship between metabolites and microbial community and function, metabolites in wheat *qu* and HJFM were measured. Wheat *qu* solid powder (10 g) was mixed with sterile water (20 mL) by rotational shaking at 150 rpm for 2 h at room temperature and then filtered through a double layer of filter paper (Whatman International, Ltd., England). The HJFM samples were also filtered through a double layer of filter paper. Both filtrates were used for the following experiments.

(1) Organic acids

Reverse-phase high-performance liquid chromatography (RP-HPLC) was used to determine quantities of the eight organic acids: α-ketoglutaric acid, pyruvate, oxalate, acetate, malate, tartrate, citrate, and lactate. Wheat *qu* and HFJM filtrates were mixed with 2 mL of trichloroacetic acid (TCA, 10%, w/v), kept at 4 °C overnight, and then centrifuged at 8000 *g* for 15 min. The supernatants were then analyzed using an RP-HPLC system (Waters e2695, Milford, MA) equipped with an Athena C18-WP column (250 × 4.6 mm; 5 μm) and a UV detector. The column temperature was maintained at 30 °C. The detection wavelength was 210 nm. The mobile phase was phosphate buffer (0.025 M NaH_2_PO_4_, pH 3.1), and the flow rate was 0.7 mL/min.

(2) Free amino acids. Wheat *qu* and HJFM were analyzed for the presence of aspartate (Asp), glutamate (Glu), serine (Ser), histidine (His), glycine (Gly), threonine (Thr), arginine (Arg), alanine (Ala), tyrosine (Tyr), cysteine (Cys), valine (Val), methionine (Met), phenylalanine (Phe), isoleucine (Ile), leucine (Leu), lysine (Lys), and proline (Pro) according to the previous methods^44^. Briefly, the filtrates were mixed with 2 mL of TCA (10%, w/v) for 4 h, and then centrifuged at 8000 *g* for 30 min. The supernatant was then analyzed using an RP-HPLC system equipped with an ODS HYPERSIL column (250 × 4.6 mm, 5 μm) and a UV detector. The column temperature was maintained at 40 °C. The detection wavelengths were 338 nm and 262 nm, and the flow rate was 1.0 mL/min.

(3) Volatile compounds. The volatile compounds were determined by using headspace solid-phase microextraction (HS-SPME) and analyzed using gas chromatography-mass spectrometry (GC-MS) according to the method reported with modifications^45–48^. The filtrates (5 mL), prepared as described above was placed in a 20-mL glass SPME vial together with 3.0 g of sodium chloride and 25 µL of 2-octanol (41.2 mg/L in absolute ethanol) as an internal standard. The vial was incubated for 50 min at 50 °C. After extraction, the fiber was introduced into the injection port of the GC-MS system (at 250 °C for 7 min) and the analytes extracted from the fiber were thermally desorbed. Mass spectra and retention indices of compounds detected by GC-MS analysis were compared with those found in published data and the MS library of the National Institute for Standards and Technology (NIST, Search 2.0). The quantity of volatile compounds was established by using the internal standard method and external standard calibration curve methods.

### Bioinformatic analysis

Sequence reads were first screened to remove low-quality reads (quality value less than 38 ≥ 40 bp, and ≥10% N containing reads) by Readfq (V8, https://github.com/cjfields/readfq). DNA sequence reads that aligned to the wheat, rice and human genomes were removed (Bowtie 2.2.4, parameters: --end-to-end, --sensitive, -I 200, -X 400). The remaining high-quality reads were assembled and analyzed using SOAP denovo software version 2.04 (parameters: -d 1, -M 3, -R, -u, -F, -K 55)^49–53^. Unused reads from each sample were assembled using the same parameters. Genes were predicted using MetaGeneMark (Version 2.10) software with default parameters^54–58^. This process produced 14.92 Gbp of raw data per sample on average. Duplicate, low quality and host reads were removed from the raw data set. For taxonomic assignment, the predicted genes were aligned to the integrated non-redundant database (NR) using DIAMOND software (Version 0.9.9) with E ≤ 1e^-5^^59^. For functional analysis, all the genes in our catalogue were aligned to the KEGG database (http://www.kegg.jp/kegg/) using DIAMOND (Version 0.9.9, default parameter except that -k 50 –sensitive -e 0.00001).

To obtain statistics of the relative abundance of different functional hierarchies, the relative abundance of each functional hierarchy was considered equal to the sum of the relative abundances annotated to that functional level. To elucidate the biosynthetic pathways responsible for the metabolites identified within *huangjiu* fermentation samples, the gene ID numbers of related enzymes, the relative abundance of corresponding enzymes, and the taxonomy annotation were extracted from the KEGG annotation. Metabolic pathways of functional microorganism were constructed by using KEGG annotations. DNA sequencing, gene prediction, assembly and annotation statistics are shown in Supplementary Table 5.

### Isolation of *Saccharopolyspora* and whole-genome sequencing of *S. hirsuta* J2

For microbial isolation on laboratory culture medium, wheat *qu* samples were suspended in sterile distilled water, and serial dilutions were plated onto Gauzeˊs Medium (Sigma) for incubation at 30 °C for between 7 to 14 days. Since members of *Saccharopolyspora* are predominant in wheat *qu* and HJFM, and were likely the core functional microorganisms in HJFM, especially *Saccharopolyspora hirsute*, Saccharopolyspora-like colonies^60^ were isolated, purified, and preserved in 30% w/v glycerol in water at −80 °C. 16 S rRNA gene sequencing using the 16S-27F and 16S-1492 primers was used for taxonomic identification^61^. The isolated strains were identified as three *S. hirsuta* (J1 and J3), two *S. rectivirgula* (J4 and J5), three *S. shandongensis* (J6 to J8), and one *S. erythraea* (J9). *S. hirsuta* J2 was deposited in China Center for Type Culture Collection under the number CCTCC M 2020103. The whole genome of *S. hirsute* J2, the strain with the highest activity of amylase, glucoamylase and protease, was sequenced using Nanopore PromethION platform (Oxford Nanopore Technologies Ltd, Oxford, UK) and Illumina Navaseq PE150 (Illumina, SanDiego, CA, USA) at the Beijing Novogene Bioinformatics Technology Co., Ltd.

### Preparation and enzyme activities of cooked wheat qu

To analyze the enzyme activities of wheat qu, that of samples from factory and artificially cooked wheat *qu* by isolated microbial strains were compared. The wheat *qu* sampled from factory was used as the control, *A. flavus* SU-16 cooked wheat qu, *A. oryzae* MQ cooked wheat *qu* and *Lactiplantibacillus plantarum* JN01 cooked wheat *qu* were used as references, and nine cooked wheat *qu* by *Saccharopolyspora* species J1 to J9 respectively were the experimental group.

To prepare the cooked wheat *qu* inoculation, wheat was prepared by crushing in a barley press roller and sterilization at 121 °C for 30 min. After cooling to <40 °C, strains of selected *A. flavus* SU-16, *A. oryzae* MQ, *L. plantarum* JN01, and *Saccharopolyspora* species J1 to J9 were inoculated (2.0 × 10^6^ cfu/g) into the sterilized wheat separately and stirred until homogeneous with additional water approximately 25% of total weight. The inoculated samples were incubated for 72 h at 28 °C (*A. flavus* SU-16 and *A. oryzae* MQ) or 37 °C (9 isolated *Saccharopolyspora* species and *L. plantarum* JN01). The final (also known as cooked) wheat *qu* was dried until its moisture content reached less than approximately 15%.

Protease, glucoamylase and amylase activities in wheat *qu* sampled from the brewery and 12 cooked wheat *qu* were determined as previously described^11^. One unit of amylase activity was defined as the amount of dry sample required for the liquefaction of 1 g starch per hour in sodium phosphate buffer (100 mM, pH 4.6) at 30 °C (U/g dry sample). One unit of glucoamylase activity was defined as the amount of dry samples required for the liberation of 1 mg glucose per hour in sodium acetate buffer (50 mM, pH 4.6) at 30 °C (U/g dry sample). One unit of protease activity was defined as the amount of dry samples required for the liberation of 1 μg of tyrosine per min in sodium acetate buffer (50 mM, pH 4.6) at 30 °C (U/g dry sample).

### Characterization of BAs metabolism by *S. hirsuta* J2

To evaluate the capacity of *S. hirsuta* J2 to produce BAs, that strain was inoculated at a level of 1 × 10^7^ cells/mL in Gauzeˊs liquid medium (Guangdong Huankai Microbial Sci. & Tech.co., Ltd, China) containing 1.0 g/L of either histidine, tyrosine, tryptophan, phenylalanine, ornithine monohydrochloride, lysine, or agmatine sulfate salt and incubated at 30 °C for 24 h. All amino acids were purchased from Sigma. Negative controls were not inoculated. BAs quantification was performed as previously described^62^ using an RP-HPLC system (Waters e2695, Milford, MA) equipped with an XBridge C18 (250 mm × 4.6 mm, 5 µm) and UV detector.

For quantification of the capacity of *S. hirsuta* J2 to consume BAs, *S. hirsuta* J2 was inoculated into Gauzeˊs liquid medium containing 50 mg/L each of histamine, tyramine, cadaverine, putrescine, spermine, spermidine, tryptamine, and phenylethylamine (each from Sigma). All cultures, including the negative control, were incubated at 30 °C for 5 days. BA consumption was measured by collecting *S. hirsuta* by centrifugation at 10,000 g for 10 min and then using the supernatant to measure BA concentrations by HPLC. *S. hirsuta J2* BA degradation was calculated according to the formula:

$${\text{BAs degradation}} (\%) = [(A-B)/A]\times 100\%$$

A was the concentration of BA in the negative controls and B was the concentration of the BA in the medium incubated with *S. hirsuta* J2.

### Application of S. hirsuta J2 in huangjiu, sausage and soy sauce fermentations

To evaluate the capability of degrading BAs in fermentation, *S. hirsuta* J2 and several other strains were used for fermentation of *Huangjiu*, sausage, and soy sauce. Notably, all the fermentation microorganisms used here do not produce biogenic amines, and biogenic amines producing in *huangjiu* fermentation is spontaneous.

(1) *Huangjiu* fermentation. Lab-scale *huangjiu* fermentations (3 L) were prepared in triplicate (*n* = 3) with *S. cerevisiae* HJ^63^ and either *S. hirsuta* J2, *A. flavus* SU-16^9^, *Aspergillus oryzae* MQ^9^, or *Lactiplantibacillus plantarum* JN01^16^ cooked wheat qu. *Huangjiu* was produced with the techniques used by a *huangjiu* brewery in Shaoxing (Zhejiang Province), China (Fig. 1). In brief, after the steamed rice was cooled to <30 °C, fermentation was initiated by adding cooked wheat *qu* (13.6 g/100 g uncooked rice), *S. cerevisiae* HJ (11.4 mL/100 g uncooked rice), and water (125 mL/100 g uncooked rice). Wheat *qu* sampled from the brewery was used as a positive control (the control group). The rice mixtures were incubated at 30 °C for 100 h and then maintained at 16 °C for 200 h. *Huangjiu* was examined for reducing sugars using the 3,5-dinitrosalicylic acid (DNS) method^64^. Titratable acidity, alcohol content, and amino acid nitrogen were measured using standard methods^65^. BAs, amino acids, organic acids, and volatile compounds were quantified as described before.

(2) Sausage fermentations*.* Sausage fermentations were performed in triplicate (*n* = 3) in laboratory following the method described by Li et al. (2019). In short, fresh pork was bought in local supermarket in Wuxi. Visible fat and connective tissues were removed from commercially purchased pork (lean meat (80%) and back fat (20%)). The meat was ground and then mixed with 0.1% (w/w) spice powder (spices, garlic, chilli, pepper, cardamom), 0.01% (w/w) sodium nitrite, 2% (w/w) salt, 0.2% (w/w) compound phosphate, 0.05% (w/w) sodium ascorbate. Sausage fermentation was spontaneous, so no microbial inoculant was used. Sausages were manufactured with *S. hirsuta* J2 (10^6^ cfu/g). Sausages without added *S. hirsuta* J2 was used as a control group. The sausages were first incubated for one day at 30 ± 0.5 °C and a relative humidity (RH) of 90%. The sausages were then incubated at 16 ± 0.5 °C with successive 24 h passages at a RH of 90%, 80% and the 75% for drying. The sausages were transferred to 14 ± 0.5 °C and a RH of between 65 to 70% for 9 days. Each sausage type was made in triplicate (*n* = 3).

(3) Soy sauce fermentation. Soy sauce was prepared in triplicate (*n* = 3) using the method described by Yang et al.^66^, with slight modifications. As above, dry soybeans were bought in local supermarket in Wuxi, and these with low quality were filtered. Cooked soybeans were mixed with roasted wheat (7:3 ratio) and then fermented for 72 h at 35 °C with *A. oryzae* (10^6^ cfu/g) or *S. hirsuta* J2 (10^6^ cfu/g) to prepare the koji, a pre-fermented products used to add flavor in fermentation. Soy sauce added *A. oryzae* was used as a control group. The koji was mixed with a brine solution (20% w/v NaCl) and fermented for 40 days at between 40 to 45 °C. The soy sauce was then prepared by pressing, filtration and pasteurization.

### Statistical analyses

The R packages ggplot2 and pheatmap were used for data visualization. A bidirectional orthogonal partial least squares (O2PLS) model was used to elucidate the association between microbial species and metabolites produced during *huangjiu* fermentation. O2PLS analysis was performed using SIMCA 14.1 software (Umetrics AB, Umeå, Sweden). Differences between the mean values for individual groups were assessed with variance tests (two-way analysis of variance (ANOVA) and one-way ANOVA) and Duncan’s multiple range tests. A value of 0.05 was set as the significance level; the data were marked as (*) *P* < 0.05, (**) *P* < 0.01, (***) *P* < 0.001, and (****) *P* < 0.0001. The *p*-value above 0.05 was considered as non-significant (ns).

### Supplementary information

Supplementary information

Supplementary Table

Reporting-summary

## Data Availability

All raw data for the current study have been deposited in National Center for Biotechnology Information (NCBI) under the accession number PRJNA694576.
