# Supplementary material for: Integrated meta-omics approaches reveal Saccharopolyspora as the core functional genus in huangjiu fermentations
Source: NPJ Biofilms Microbiomes. 2023 Sep 19;9:65. doi: 10.1038/s41522-023-00432-1 (PMC10509236; doi:10.1038/s41522-023-00432-1)
Supplement: Supplementary file 1 — Supplementary information [file 41522_2023_432_MOESM1_ESM.pdf]

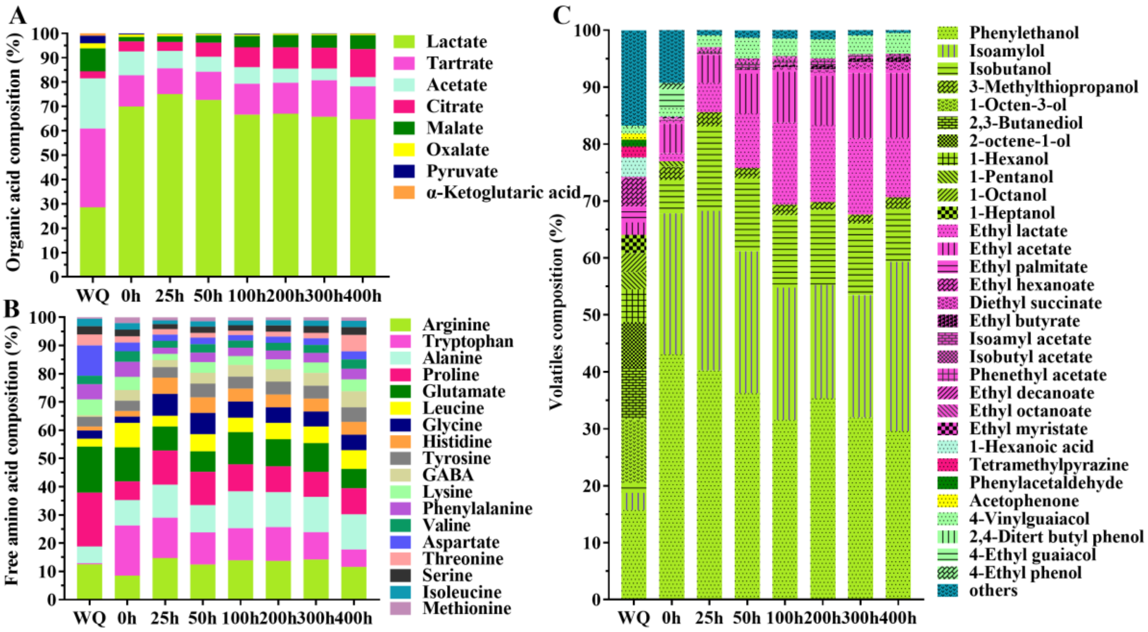

Supplementary Figure 1 Dynamic of the metabolites during *huangjiu* fermentation process. The relative abundance of organic acids (A), amino acids (B), and 31 dominant volatile compounds (C).

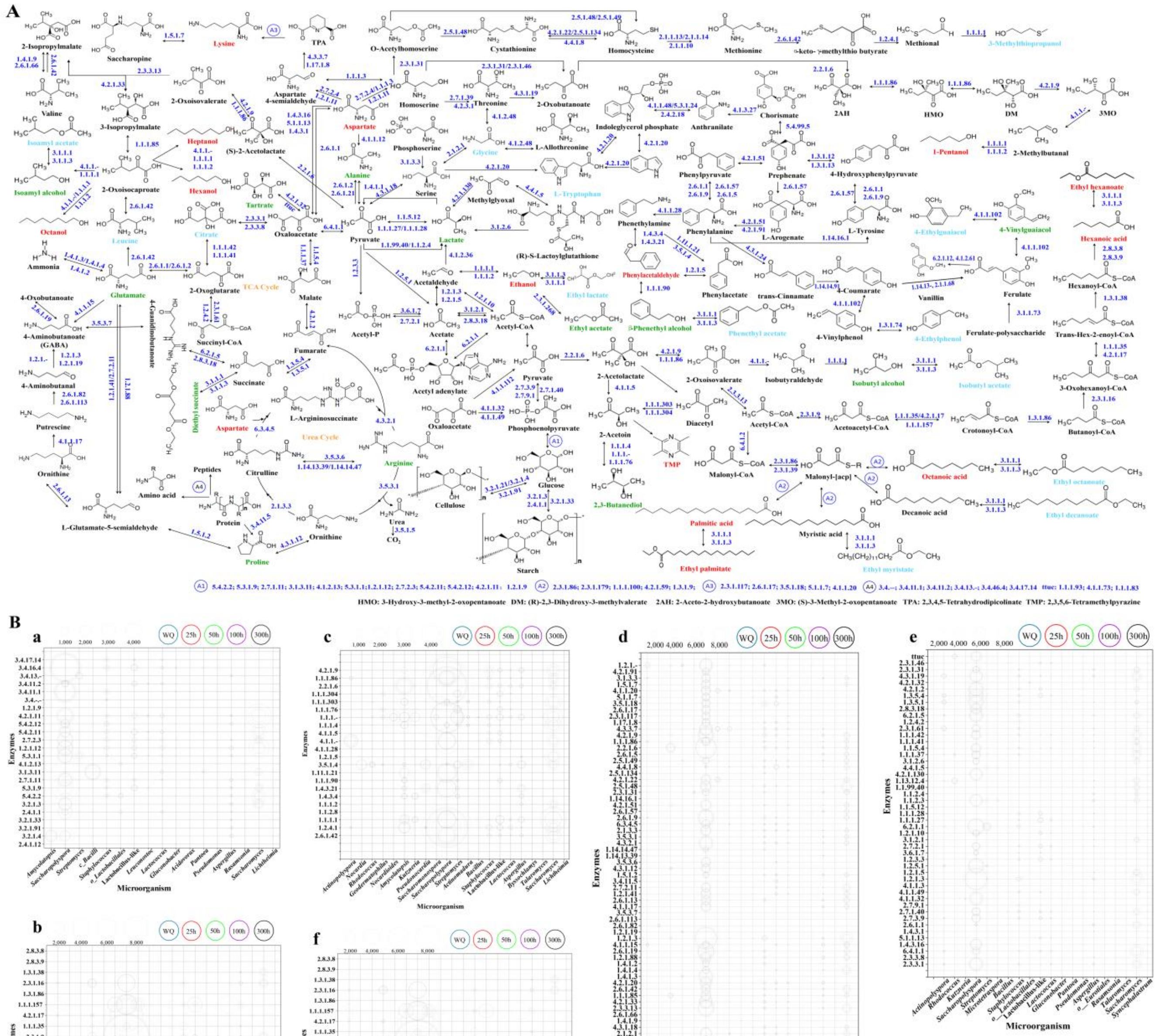

C

1.1.1.1	Alcohol dehydrogenase	1.2.1.11	Aspartate-semialdehyde dehydrogenase	1.5.1.7	Lysine-2-oxoglutarate reductase	2.6.1.21	D-Amino-acid transaminase	3.2.1.91	Cellulose 1,4-beta-cellobiosidase	4.2.1.32	Tartrate dehydratase
1.1.1.100	Beta-ketoacyl reductase	1.2.1.12	Triosephosphate dehydrogenase	2.1.1.10	Homocysteine S-methyltransferase	2.6.1.42	Branched-chain-amino-acid transaminase	3.4.11.5	Prolyl aminopeptidase	4.2.1.33	3-Isopropylmalate dehydratase
1.1.1.157	3-Hydroxybutyryl-CoA dehydrogenase;	1.2.1.19	Aminobutyraldehyde dehydrogenase	2.1.1.13	Methionine synthase	2.6.1.5	Tyrosine transaminase	3.5.1.18	Succinyl-diaminopimelate desuccinylase	4.2.1.51	Prephenate dehydratase
1.1.1.2	Alcohol dehydrogenase (NADP+)	1.2.1.3	Aldehyde dehydrogenase (NAD+)	2.1.1.14	Homocysteine methylase	2.6.1.57	Aromatic-amino-acid transaminase	3.5.1.4	Amidase	4.2.1.59	3-Hydroxyacyl-[acyl-carrier-protein] dehydratase
1.1.1.27	L-lactate dehydrogenase	1.2.1.41	Glutamate-5-semialdehyde dehydrogenase	2.1.2.1	Glycine hydroxymethyltransferase	2.6.1.66	Valine pyruvate transaminase	3.5.1.5	Urease	4.2.1.9	Dihydroxy-acid dehydratase
1.1.1.28	D-lactate dehydrogenase	1.2.1.5	Aldehyde dehydrogenase [NAD(P)+]	2.1.3.3	Ornithine carbamoyltransferas	2.6.1.82	Putrescine-2-oxoglutarate transaminase	3.5.3.1	Arginase	4.2.1.91	Arogenate dehydratase
1.1.1.3	Homoserine dehydrogenase	1.2.1.88	L-glutamate gamma-semialdehyde dehydrogenase	2.2.1.6	Acetolactate synthase	2.6.1.9	Histidinol-phosphate transaminase	3.5.3.6	Arginine deiminase	4.2.3.1	Threonine synthase
1.1.1.303	Diacetyl reductase [(R)-acetoin forming]	1.2.1.9	Glyceraldehyde phosphate dehydrogenase (NADP+)	2.3.1.117	Tetrahydropicolinate succinylase	2.7.1.11	Phosphohexokinase	3.5.3.7	Guanidinobutyrase	4.3.1.12	Ornithine cyclodeaminase
1.1.1.304	Diacetyl reductase [(S)-acetoin forming]	1.2.3.3	Pyruvate oxidase	2.3.1.16	Acetyl-CoA C-acyltransferase	2.7.1.39	Homoserine kinas	3.6.1.7	Acylphosphatase	4.3.1.18	D-serine ammonia-lyase
1.1.1.35	3-Hydroxyacyl-CoA dehydrogenase;	1.2.4.1	Pyruvate dehydrogenase (Acetyl-transferring)	2.3.1.179	3-Oxoacyl-acyl carrier protein synthase I	2.7.1.40	Pyruvate kinase	4.1.1.102	Phenacrylate decarboxylase	4.3.1.19	Threonine ammonia-lyase
1.1.1.37	Malate dehydrogenase	1.2.4.2	Oxoglutarate dehydrogenase (Succinyl-transferring)	2.3.1.31	Homoserine O-acetyltransferase	2.7.2.1	Acetate kinase	4.1.1.112	Oxaloacetate decarboxylase	4.3.1.24	Phenylalanine deaminase
1.1.1.4	(R,R)-butanediol dehydrogenase	1.2.5.1	Pyruvate dehydrogenase (Quinone)	2.3.1.39	Malonyl transacylase	2.7.2.11	Glutamate 5-kinase	4.1.1.12	Aspartate 4-decarboxylase	4.3.2.1	Argininosuccinate lyase
1.1.1.41	(R,R)-butanediol dehydrogenase	1.3.1.12	Prephenate dehydrogenase	2.3.1.46	Homoserine O-succinyltransferase	2.7.2.3	Phosphoglycerate kinase	4.1.1.15	Glutamate decarboxylase	4.3.3.7	4-Hydroxy-tetrahydrodipicolinate synthase
1.1.1.42	Isocitrate dehydrogenase (NADP+)	1.3.1.13	Prephenate dehydrogenase (NADP+)	2.3.1.61	Dihydrolipoyllysine-residue succinyltransferase	2.7.2.4	Aspartate kinase	4.1.1.17	Ornithine decarboxylase	4.4.1.5	Lactoylglutathione lyase
1.1.1.76	(S,S)-butanediol dehydrogenase	1.3.1.38	Trans-2-enoyl-CoA reductase (NADPH)	2.3.1.86	Fatty-acyl-CoA synthase system	2.7.3.9	Phosphoenolpyruvate-protein phosphotransferase	4.1.1.20	Diaminopimelate decarboxylase	4.4.1.8	Lyases
1.1.1.85	3-Isopropylmalate dehydrogenase	1.3.1.74	2-Alkenal reductase	2.3.1.9	Acetyl-CoA C-acetyltransferase;	2.7.9.1	Pyruvate-phosphate dikinase	4.1.1.28	Aromatic-L-amino-acid decarboxylase	5.1.1.13	Aspartate racemase
1.1.1.86	Ketol-acid reductoisomerase (NADP+)	1.3.1.86	Crotonyl-CoA reductase	2.3.3.1	2-Isopropylmalate synthase	2.8.3.18	Succinyl-CoA:acetate CoA-transferase	4.1.1.32	Phosphoenolpyruvate carboxykinase (GTP)	5.1.1.7	Diaminopimelate epimerase
1.1.1.90	Aryl-alcohol dehydrogenase	1.3.1.9	Enoyl-ACP reductase	2.3.3.13	Citrate (Si)-synthase	2.8.3.8	Acetate CoA-transferase	4.1.1.48	Indoleglycerol phosphate synthetase	5.3.1.1	Triose-phosphate isomerase
1.1.2.3	L-Lactate dehydrogenase (Cytochrome)	1.3.5.1	Fumarate reductase (Quinol)	2.3.3.8	ATP citrate synthase	2.8.3.9	Butyrate--acetoacetate CoA-transferase	4.1.1.49	Phosphoenolpyruvate carboxykinase (ATP)	5.3.1.24	Phosphoribosylanthranilate isomerase
1.1.2.4	D-Lactate dehydrogenase (Cytochrome)	1.3.5.4	Succinate dehydrogenase	2.4.1.1	Glycogen or starch phosphorylase	3.1.1.1	Carboxylesterase	4.1.1.5	Acetolactate decarboxylase	5.3.1.9	Phosphohexose isomerase
1.1.2.8	Alcohol dehydrogenase (Cytochrome C)	1.4.1.1	Alanine dehydrogenase	2.4.2.18	Anthranilate phosphoribosyltransferase	3.1.1.3	Triacylglycerol lipase	4.1.2.13	Aldolase	5.4.2.11	Phosphoglycerate mutase
1.1.5.12	D-Lactate dehydrogenase (Quinone)	1.4.1.2	Glutamate dehydrogenase	2.5.1.134	Cystathionine beta-synthase (O-acetyl-L-serine)	3.1.1.73	Feruloyl esterase	4.1.2.36	Lactate synthase	5.4.2.12	Phosphoglycerate mutase
1.1.5.4	Malate dehydrogenase (Quinone)	1.4.1.3	Glutamate dehydrogenase [NAD(P)+]	2.5.1.48	Cystathionine gamma-synthase	3.1.2.1	Acetyl-CoA hydrolase	4.1.2.48	Low-specificity L-threonine aldolase	5.4.2.2	Glucose phosphomutase
1.1.99.40	(R)-2-hydroxyglutarate-pyruvate transhydrogenase	1.4.1.4	Glutamate dehydrogenase (NADP+)	2.5.1.49	O-acetylhomoserine aminocarboxypropyltransferase	3.1.2.6	Hydroxyacylglutathione hydrolase	4.1.3.27	Anthranilate synthase	5.4.99.5	Chorismate mutase
1.11.1.21	Malate dehydrogenase (Quinone)	1.4.1.9	Leucine dehydrogenase	2.6.1.1	Aspartate transaminase	3.1.3.11	Hexose diphosphatase	4.2.1.11	Phosphopyruvate hydratase	6.2.1.1	Acetate-CoA ligase
1.14.13.39	Nitric-oxide synthase (NADPH)	1.4.3.1	D-aspartate oxidase	2.6.1.113	Putrescine--pyruvate transaminase	3.1.3.3	Phosphoserine phosphatase	4.2.1.130	D-lactate dehydratase	6.2.1.5	Succinate-CoA ligase (ADP-forming)
1.14.14.47	Nitric oxide synthetase	1.4.3.16	L-aspartate oxidase	2.6.1.13	Ornithine aminotransferase	3.2.1.21	beta-glucosidase	4.2.1.17	enoyl-CoA hydratase;	6.3.4.5	Argininosuccinate synthase
1.14.16.1	Phenylalanine 4-monooxygenase	1.4.3.21	Primary-amine oxidase	2.6.1.17	Succinyldiaminopimelate transaminase	3.2.1.3	Glucoamylase	4.2.1.2	Fumarate hydratase	6.4.1.1	Pyruvate carboxylase
1.17.1.8	4-Hydroxy-tetrahydrodipicolinate reductase	1.4.3.4	Monoamine oxidase	2.6.1.19	4-Aminobutyrate--2-oxoglutarate transaminase	3.2.1.33	Amylo-alpha-1,6-glucosidase	4.2.1.20	Tryptophan synthase	6.4.1.2	**Acetyl**-CoA carboxylase
**1.2.1.10**	Acetaldehyde dehydrogenase (Acetylating)	**1.5.1.2**	Pyrroline-5-carboxylate reductase	**2.6.1.2**	Alanine transaminase	**3.2.1.4**	Cellulase	**4.2.1.22**	Cystathionine beta-synthase	ttuc	Tartrate dehydrogenase/decarboxylase

Supplementary Figure 2 Predicted metabolic network for the production of the

dominant metabolites by the microbial community of wheat *qu* and *huangjiu*. Metabolites in red are unique to wheat *qu*; metabolites in blue are unique to HJFM; metabolites in green are found in both wheat *qu* and HJFM. The metabolic networks predicted to produce these metabolites are shown in Figure 2A. The relationship between microorganisms and enzymes involved in different metabolic pathways are shown in Figure 2B, in which a–f was correlated with raw material degradation, esters biosynthesis, alcohols biosynthesis, amino acids biosynthesis, organic acids biosynthesis and others, respectively. The enzyme names are shown in Figure 2C.

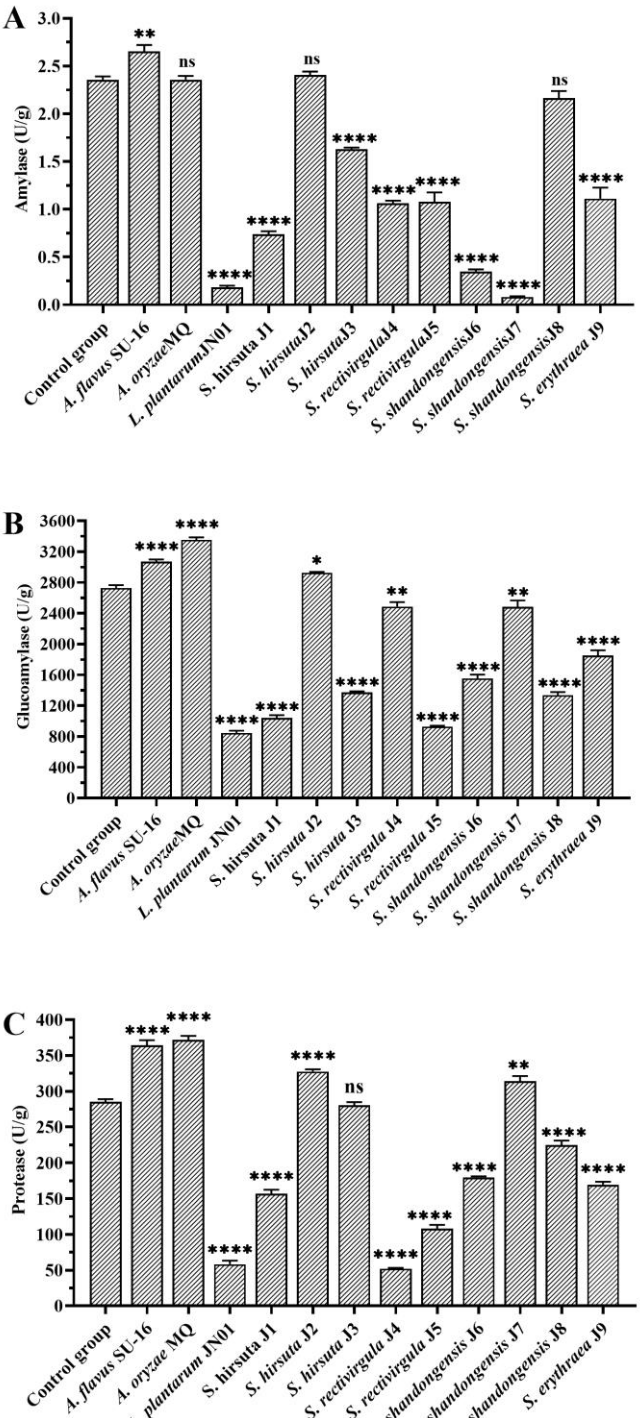

Supplementary Figure 3 Enzymatic activities of *qu* inoculated with *Aspergillus*, *Saccharopolyspora* or *L. plantarum*. A value of 0.05 was set as the significance level compared with Control group; the data were marked as (*) $p < 0.05$, (**) $p < 0.01$, (***) $p < 0.001$, and (****) $p < 0.0001$. The p-value above 0.05 was considered as non-significant (ns). Error bars in histogram mead the standard deviation of observed data

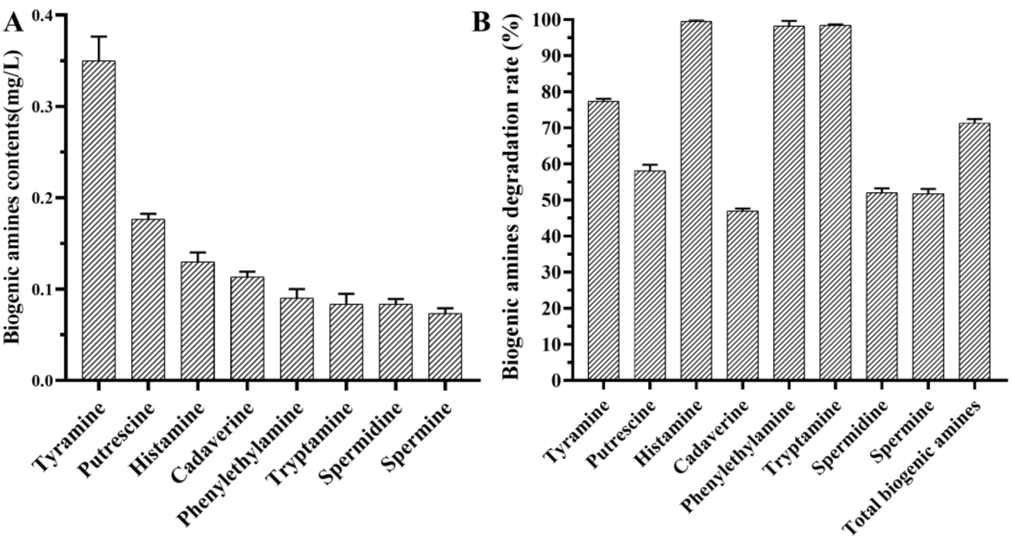

Supplementary Figure 4 BAs production (A) and degradation abilities (B) of *S. hirsuta* J2 in Gauze´s liquid medium after 24 h. Error bars in histogram mead the standard deviation of observed data.
